# Prognosis of tumor infiltrating lymphocytes in operable tongue cancer patients

**DOI:** 10.1186/2051-1426-2-S3-P198

**Published:** 2014-11-06

**Authors:** Wan-Yu Chen, Yih-Leong Chang, Sung-Hsin Kuo, Ann-Lii Cheng

**Affiliations:** 1 Division of Radiation Oncology, Department of Oncology, National Taiwan University Hospital, Taipei, Taiwan; 2 Department of Pathology, National Taiwan University Hospital and National Taiwan University College of Medicine, Taipei, Taiwan; 3 Department of Oncology, National Taiwan University Hospital, Taipei, Taiwan

## Background

The immune microenvironment is important to the pathophysiology of head and neck squamous cell carcinoma (HNSCC). Our aim was to investigate the prognostic significance of tumour-infiltrating lymphocytes (TILs) in operable tongue cancer patients treated with curative surgery and adjuvant radiotherapy with or without chemotherapy.

## Patients and methods

The presence of CD3+, CD4+, CD8+ and FOXP3+ TILs in tumor tissues obtained from 93 patients during surgery were examined by immunohistochemistry. Correlation between clinicopathological features and TILs was investigated. The prognostic roles of TILs for local recurrence-free survival (LRFS), regional recurrence-free survival (RRFS), distant metastasis-free survival (DMFS) and overall survival (OS) were analyzed.

## Results

**Figure 1:**
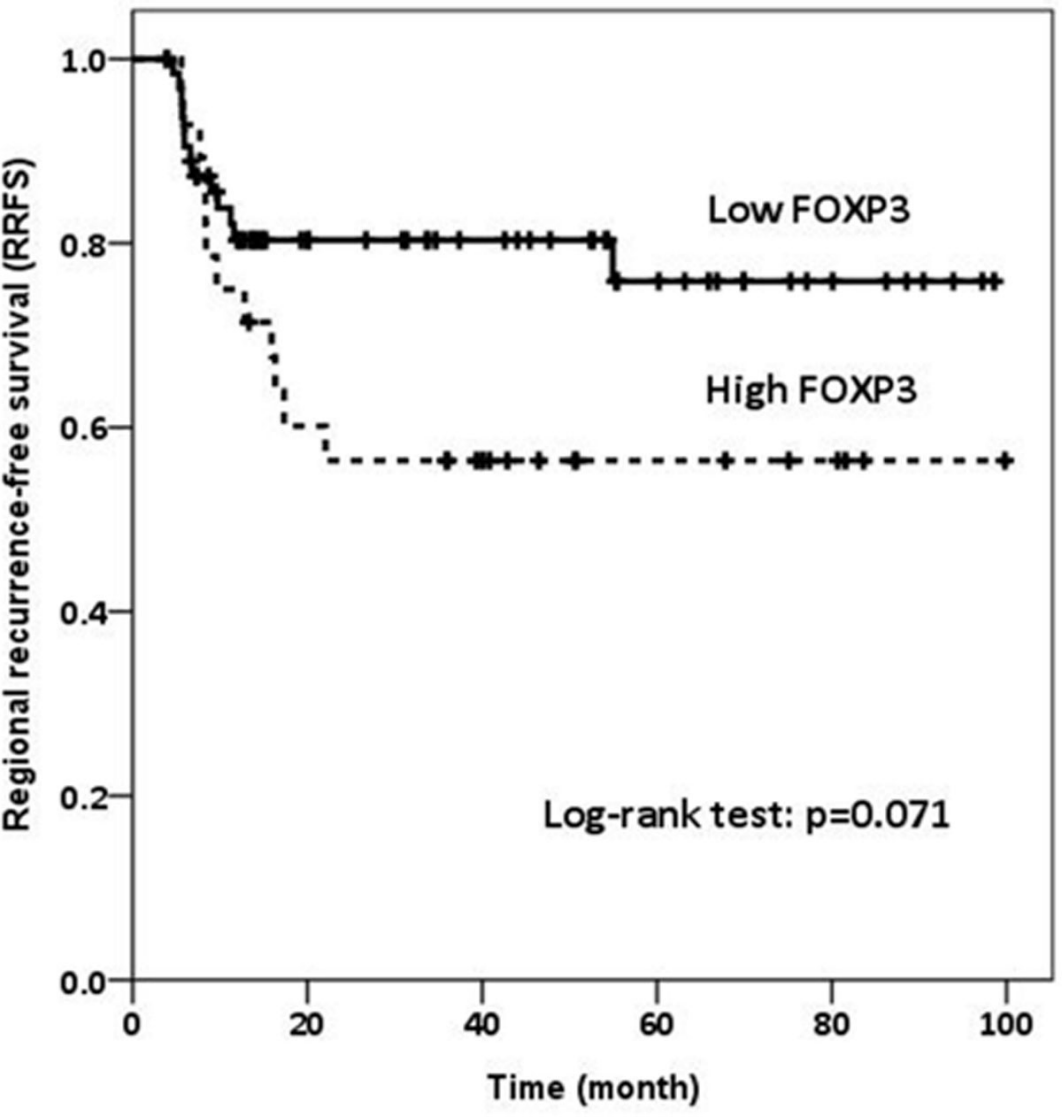
**Regional recurrence-free survival (RRFS) according to FOXP3+ TILs**.

**Figure 2:**
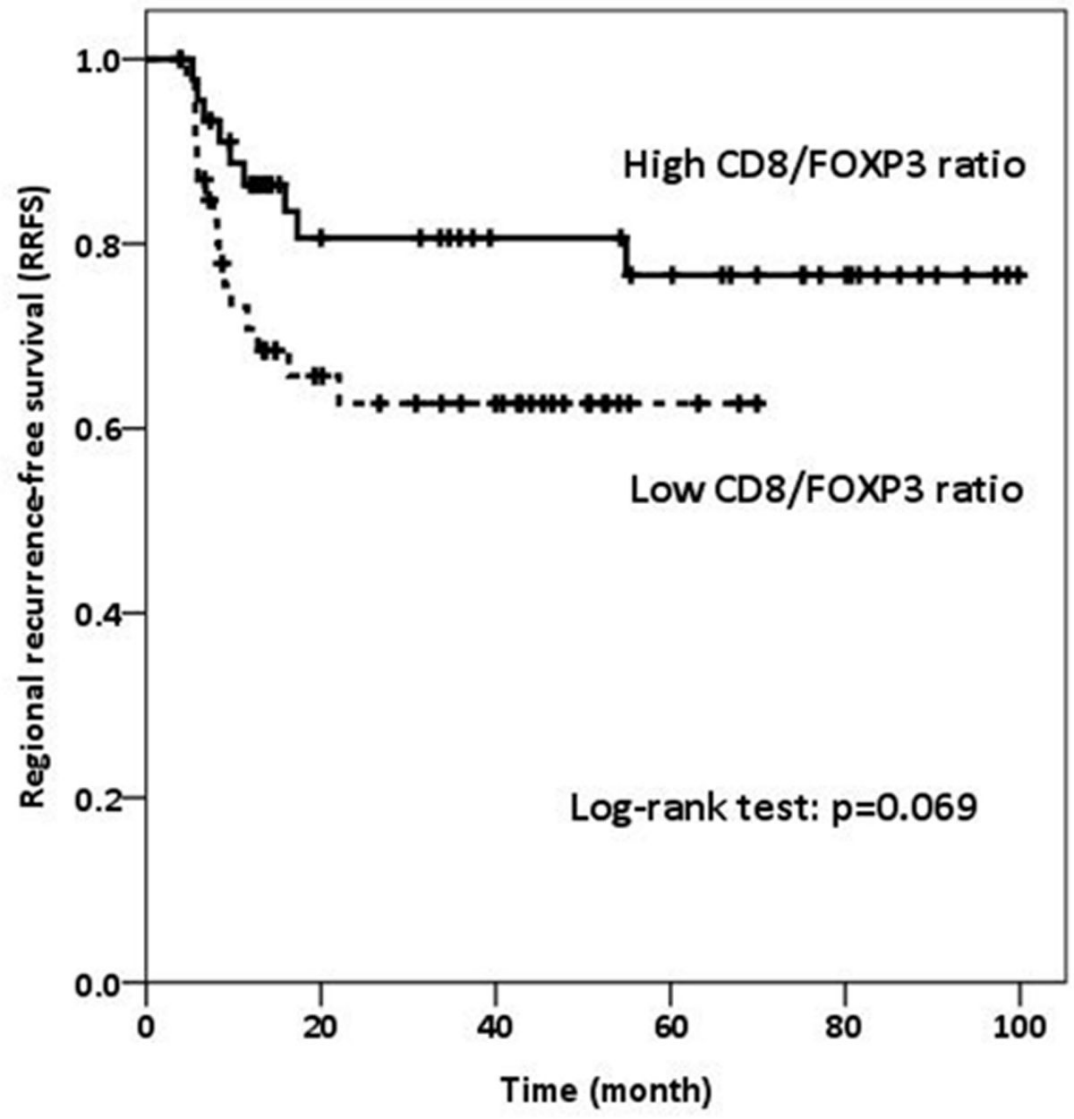
**RRFS according to CD8/FOXP3 ratio**.

**Figure 3:**
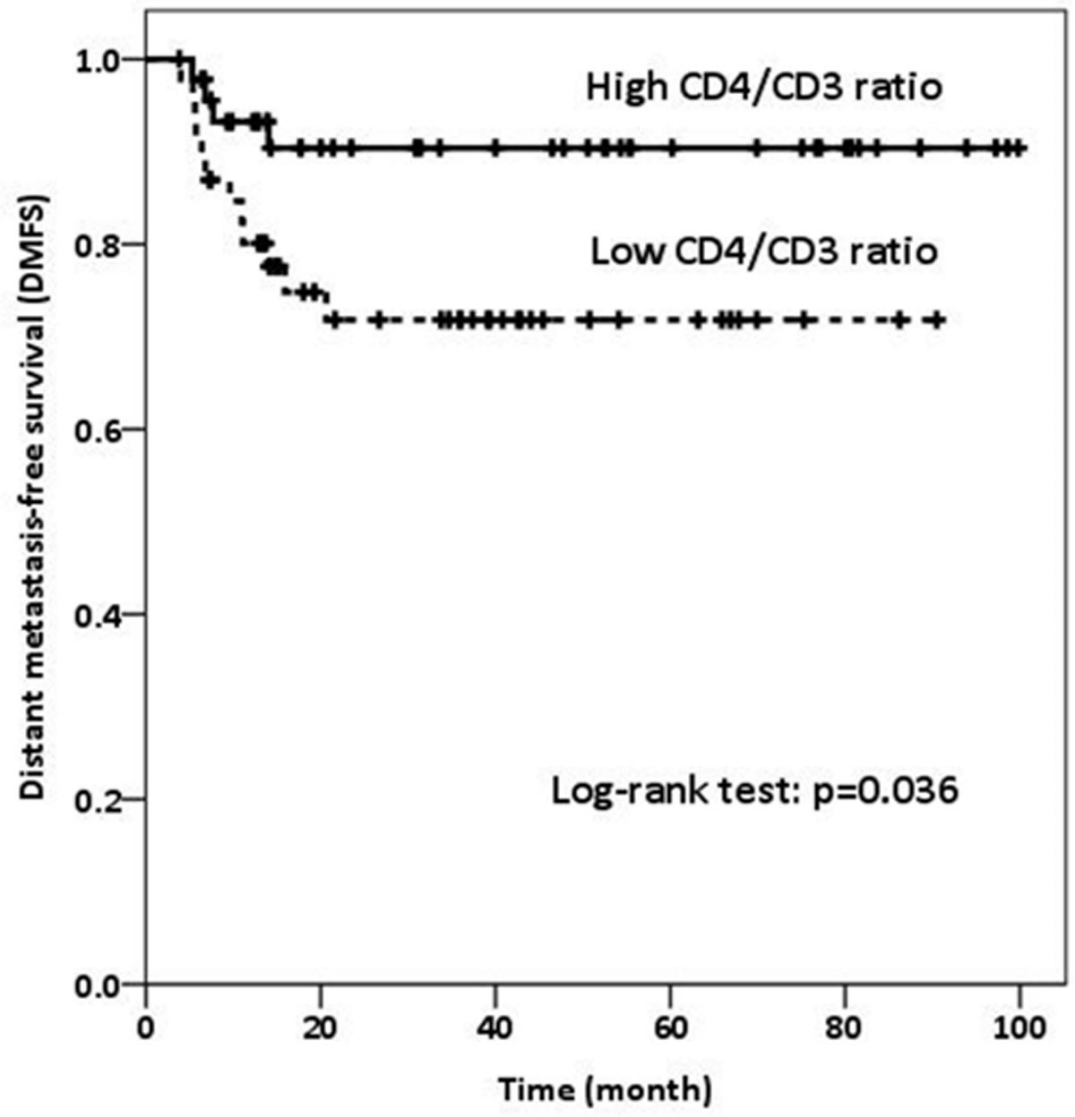
**Distant metastasis-free survival (DMFS) according to CD4/CD3 ratio**.

Median follow up time was 31.4 months (range, 0.2-99.8 months). Higher number of CD4+ cells (p = 0.006), higher CD4/FOXP3 ratio (p = 0.012), lower CD3/CD4 ratio (p = 0.043), and higher CD4/CD8 ratio (p = 0.006) were correlated with the absence of lymphovascularinvasion (LVI). Patients with lower FOXP3+ TILs and higher CD8/FOXP3 ratio had marginally better RRFS (p = 0.071, and p = 0.069, respectively) (Figure 1 and Figure 2.). Patients with higher CD4/CD3 ratio had a significantly better DMFS (p = 0.036) (Figure 3).

## Conclusion

CD4+ TILs and its ratio to other TILs were inversely correlated with LVI. Higher CD4/CD3 ratio predicts better DMFS. Prognostic role of FOXP3 in RRFS was marginally significant and warrants further investigation.

